# Proposed diagnostic volumetric bone mineral density thresholds for osteoporosis and osteopenia at the cervicothoracic spine in correlation to the lumbar spine

**DOI:** 10.1007/s00330-022-08721-7

**Published:** 2022-04-06

**Authors:** Sebastian Rühling, Andreas Scharr, Nico Sollmann, Maria Wostrack, Maximilian T. Löffler, Bjoern Menze, Anjany Sekuboyina, Malek El Husseini, Rickmer Braren, Claus Zimmer, Jan S. Kirschke

**Affiliations:** 1 Department of Neuroradiology, School of Medicine, Klinikum rechts der Isar, Technical University of Munich, 81675 Munich, Germany; 2 TUM-Neuroimaging Center, Klinikum rechts der Isar, Technical University of Munich, Munich, Germany; 3 Department of Diagnostic and Interventional Radiology, University Hospital Ulm, Ulm, Germany; 4 Department of Neurosurgery, Klinikum rechts der Isar, School of Medicine, Technical University of Munich, Munich, Germany; 5 Department of Diagnostic and Interventional Radiology, University Medical Center Freiburg, Freiburg im Breisgau, Germany; 6 Department of Informatics, Technical University of Munich, Munich, Germany; 7 Department of Quantitative Biomedicine, University of Zurich, Zurich, Switzerland; 8 Department of Diagnostic and Interventional Radiology, School of Medicine, Klinikum rechts der Isar, Technical University of Munich, Munich, Germany

**Keywords:** Bone density, Osteoporosis, Multidetector computed tomography, Machine learning, Screening

## Abstract

**Objectives:**

To determine the correlation between cervicothoracic and lumbar volumetric bone mineral density (vBMD) in an average cohort of adults and to identify specific diagnostic thresholds for the cervicothoracic spine on the individual subject level.

**Methods:**

In this HIPPA–compliant study, we retrospectively included 260 patients (59.7 ± 18.3 years, 105 women), who received a contrast-enhanced or non-contrast-enhanced CT scan. vBMD was extracted using an automated pipeline (https://anduin.bonescreen.de). The association of vBMD between each vertebra spanning C2–T12 and the averaged values at the lumbar spine (L1–L3) was analyzed before and after semiquantitative assessment of fracture status and degeneration, and respective vertebra-specific cut-off values for osteoporosis were calculated using linear regression.

**Results:**

In both women and men, trabecular vBMD decreased with age in the cervical, thoracic, and lumbar regions. vBMD values of cervicothoracic vertebrae showed strong correlations with lumbar vertebrae (L1–L3), with a median Pearson value of *r* = 0.87 (range: *r*_C2_ = 0.76 to *r*_T12_ = 0.96). The correlation coefficients were significantly lower (*p* < 0.0001) without excluding fractured and degenerated vertebrae, median *r* = 0.82 (range: *r*_C2_ = 0.69 to *r*_T12_ = 0.93). Respective cut-off values for osteoporosis peaked at C4 (209.2 mg/ml) and decreased to 83.8 mg/ml at T12.

**Conclusion:**

Our data show a high correlation between clinically used mean L1–L3 values and vBMD values elsewhere in the spine, independent of age. The proposed cut-off values for the cervicothoracic spine therefore may allow the determination of low bone mass even in clinical cases where only parts of the spine are imaged.

**Key Points:**

- *vBMD of all cervicothoracic vertebrae showed strong correlation with lumbar vertebrae (L1–L3), with a median Pearson’s correlation coefficient of r = 0.87 (range: r*_*C2*_ *= 0.76 to r*_*T12*_ *= 0.96).*
- *The correlation coefficients were significantly lower (p < 0.0001) without excluding fractured and moderate to severely degenerated vertebrae, median r = 0.82 (range: r*_*C2*_ *= 0.69 to r*_*T12*_ *= 0.93).*
- *We postulate that trabecular vBMD < 200 mg/ml for the cervical spine and < 100 mg/ml for the thoracic spine are strong indicators of osteoporosis, similar to < 80 mg/ml at the lumbar spine.*

**Supplementary Information:**

The online version contains supplementary material available at 10.1007/s00330-022-08721-7.

## Introduction

Opportunistic measurements derived from multidetector computed tomography (MDCT) scans have become an established and well-accepted method [1, 2]. Alongside the extraction of various biometric data (e.g., quantification of liver fat or muscle density), this technique allows noninvasive assessment of bone mineral density (BMD) [3–6].

CT is a very commonly used technique and the number of CT examinations has steadily increased, whereas the numbers for dual-energy X-ray absorptiometry (DXA), the gold standard for BMD assessment, have remained low at best [7, 8]. In fact, a significant decline in DXA screening numbers and the provision of DXA services has been observed in the USA over the past two decades [9–11]. This contrasts with approximately 43.3 million people with low BMD at high risk for osteoporosis, who would benefit from appropriate screening methods [12]. Furthermore, several advantages of opportunistic BMD measurements have been described. Opportunistic CT is capable of assessing the true three-dimensional bone architecture (volumetric density), whereas DXA as a planar technique can only measure BMD per area (area density). Therefore, DXA is prone to substantial errors attributable to degenerative changes (e.g., osteophytes), vertebra size, and variations in surrounding tissue [13, 14]. Most importantly, the ability of DXA to correctly identify individuals with osteoporosis is relatively low, and in recent literature, opportunistic CT has even outperformed DXA [15, 16]. This argues for opportunistic CT as a valid alternative to accurately identify individuals with low BMD, leading to appropriate and early treatment.

Although potential cut-off values and BMD variations for the lumbar spine are well-documented, less is known about possible diagnostic thresholds for the cervicothoracic spine [2, 17, 18]. Several studies have suggested substantial BMD differences with nonsignificant correlations between different spinal regions, making it challenging to establish cut-off values in a clinically useful manner [19–22]. Hence, in clinical practice, CT scans that cover only a part of the cervical or thoracic spine restrict wider application of opportunistic BMD measurements. For example, particularly in the emergency setting (e.g., in patients with suspected stroke or traumatic brain injury), often only the cervical spine is additionally imaged, which is not used for opportunistic assessment of osteopenia and osteoporosis. Consequently, a considerable amount of data remains unused, although it could already be analyzed prospectively and retrospectively by automated pipelines [16, 23, 24].

Thus, the purpose of this study was to (1) determine the correlation between cervicothoracic volumetric BMD (vBMD) and lumbar vBMD as derived from MDCT in an average cohort of adults, and (2) to identify possible vBMD thresholds for the cervicothoracic spine on the individual subject level.

## Methods

### Study population

The local institutional review board approved this HIPPA–compliant retrospective study and waived the requirement for written informed consent. CT images were retrospectively selected from our digital picture archiving and communication system (Sectra AB). We included 260 patients that received a contrast-enhanced or non-contrast MDCT scan of at least the thoracolumbar spine at our radiology department between January 2007 and November 2019. The indication for MDCT was known or suspected trauma in most cases. Exclusion criteria were inadequate image quality (e.g., due to artifacts) and contrast application for another scan within 6 h prior to the selected scan (*n* = 41). The final dataset consisted of 260 adults (105 women and 155 men), with a mean age of 59.7 ± 18.3 years (range: 18 to 96 years, Table 1). In 212 patients, CT scans additionally included the cervical spine, resulting in a total number of 4874 vertebrae (Table 1).

**Table 1:** Characteristics of CT scans and patients

Study set	
Patients	
No. of patients	260
No. of women	105
Age (in years)^†^	59.7 ± 18.3
Imaging	
No. of scans	260
No. of cervical spines	212
No. of vertebrae	4874
No. of contrast-enhanced scans	46
No. of fractures (Genant grades 1–3)	158
No. of vertebrae (moderate to severe degenerative changes)	530
No. of patients aged < 50	73 (21*)
No. of patients aged 50–59	49 (20*)
No. of patients aged 60–69	63 (23*)
No. of patients aged > 70	75 (41*)
Scanner	
Philips lqon	29
Philips Brilliance 64	3
Philips iCT	26
Siemens Definition AS+	85
Siemens Definition AS	57
Siemens Sensation Cardiac 64	11
Siemens Biograph 128	23
Siemens Biograph 64	26

Note: Unless otherwise indicated, data are numbers of patients

^*^Number of women in this particular age group

^†^Data are means ± standard deviations

### CT imaging and data processing

**Fig. 1:**
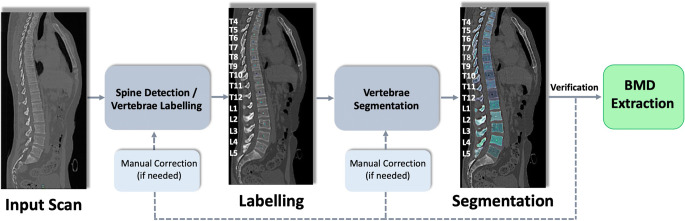
Overview of the automated spine processing and BMD extraction pipeline. Anduin (https://anduin.bonescreen.de) is used to localize, label, and segment the vertebrae. The correct labeling and segmentation of the vertebrae are verified by a neuroradiologist and manually corrected if needed. In a final step, trabecular vBMD values are automatically extracted for each vertebra that is fully depicted on the scan

CT scans were acquired with 8 different MDCT scanners from 2 different vendors using the standard clinical protocol (Table 1). Forty-six patients received standardized intravenous administration of contrast agent (Iomeron 400; Bracco). Images were acquired in a helical mode with a peak tube voltage of 120 kVp, axial slice thickness of 0.9–2 mm, and adaptive tube load. CT data were converted into Neuroimaging Informatics Technology Initiative format and reduced to a maximum of 1 mm isotropic spatial resolution. An offline version of the freely available web tool Anduin (https://anduin.bonescreen.de, Fig. 1) was used for automated spine processing and vBMD extraction. First, a low-spatial-resolution 3D artificial neural network created Gaussian heat maps and extracted bounding boxes around the spine, allowing the extraction of localized maximum intensity projections (MIPs) to locate the spine. Second, a 2D Btrfly Net was applied on the coronal and sagittal MIPs for vertebra labeling [25, 26]. The correct labeling of the vertebrae was verified by a neuroradiologist and manually corrected if needed. Third, segmentation masks were created around vertebral labels using a 3D U-Net [27, 28]. The segmentation was also reviewed by a neuroradiologist and corrected if necessary. Fourth, another 3D U-Net was used to divide segmentations into vertebral subregions, including posterior elements as well as cortical shell and trabecular compartment of the vertebral bodies.

### Evaluation of vertebrae and vBMD extraction

All CT scans were screened for fractures using a semiquantitative approach according to Genant et al [29]. Vertebrae were graded into non-fractured (grade 0) and fractured according to height loss (grade 1, 20–25%; grade 2, 25%–40%; and grade 3, ≥ 40%). Abnormal morphometry related to developmental changes, like in Scheuermann disease or in degenerative spondylarthropathy, was not rated as a fracture. Vertebrae that had a fracture grade ≥ 1 were excluded from further vBMD assessment (*n* = 158).

Degenerative changes (e.g., osteophytes or sclerosis) are known to represent a major source of accuracy errors in BMD measurements [30]. Therefore, in a second step, all scans were manually reviewed for degenerative changes. Semiquantitative screening for both fractures and degenerative changes was performed by a neuroradiologist. Vertebrae were categorized into no degenerative changes present (grade 0) and mild to severe degenerative changes (grade 1, grade 2, and grade 3). All vertebrae that were assigned a degeneration grade ≥ 2 were excluded from further vBMD assessment (*n* = 530). BMD values were automatically extracted from the segmentation masks of the trabecular compartment of vertebral bodies, and scanner-specific HU-to-BMD conversion equations previously calculated with density reference phantoms were applied [16]. Contrast-induced bias was automatically corrected by linear regression for the respective contrast phase. The extracted vBMD values were averaged over non-fractured lumbar vertebrae L1–L3.

### Statistical analysis

Statistical analyses were performed using Prism 8 (Version 9.0.0, 2020, GraphPad Software), and *p* values < 0.05 were considered statistically significant. Standard descriptive statistics were calculated for the study set. Fifty-six patients were additionally matched by age. Paired and unpaired *t* tests were used for comparisons between groups. The relationship between vBMD of each vertebra with the lumbar region (averaged values from L1 to L3) was determined using Pearson’s correlation coefficients. First, all fractured and degenerated vertebrae were excluded from the analysis. The calculation was then repeated a second time, including all fractured and degenerated vertebrae. To estimate diagnostic cut-off values for the cervicothoracic spine, linear regression between each vertebral level with the lumbar region was used. Diagnostic thresholds proposed by the American College of Radiology were applied to the lumbar spine (osteoporosis: trabecular vBMD < 80 mg/ml) [18].

## Results

Overall, 60 out of the 260 included patients had a vertebral fracture, with a total number of 158 fractured vertebrae (Genant grades 1–3). Most fractures occurred in the thoracic spine 103 (65%) and lumbar spine 53 (34%), compared to only one cervical fracture (1%).

The vBMD values (L1–L3) of patients presenting with a vertebral fracture were significantly lower compared to those without a fracture (111.7 vs. 80.0 mg/ml, *p* < 0.0001). In an age-matched cohort (*n* = 56), a significant difference (*p* = 0.02) in mean vBMD was found between women (131.3 ± 84.2 mg/ml) and men (155.5 ± 54.8 mg/ml). For both genders, vBMD was highest at C4. In the younger-age group (< 50 years), vBMD at C4 was 304.0 ± 74.8 mg/ml for women and 290.4 ± 59.8 mg/ml for men. In the older-age group (> 50 years), vBMD was 189.0 ± 67.2 mg/ml for women and 236.3 ± 62.7 mg/ml for men. The vBMD decreased from the cervical to the lumbar region.

**Fig. 2:**
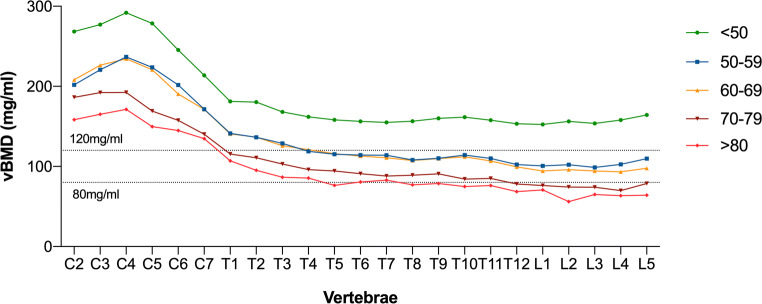
Mean vBMD for each vertebra for five different age groups (< 50, 50–59, 60–69, 70–79, and > 80 years) in women and men. The two dotted lines indicate the vBMD range between normal (vBMD > 120 mg/ml) and osteoporosis (vBMD < 80 mg/ml) as defined by the American College of Radiology

**Fig. 3:**
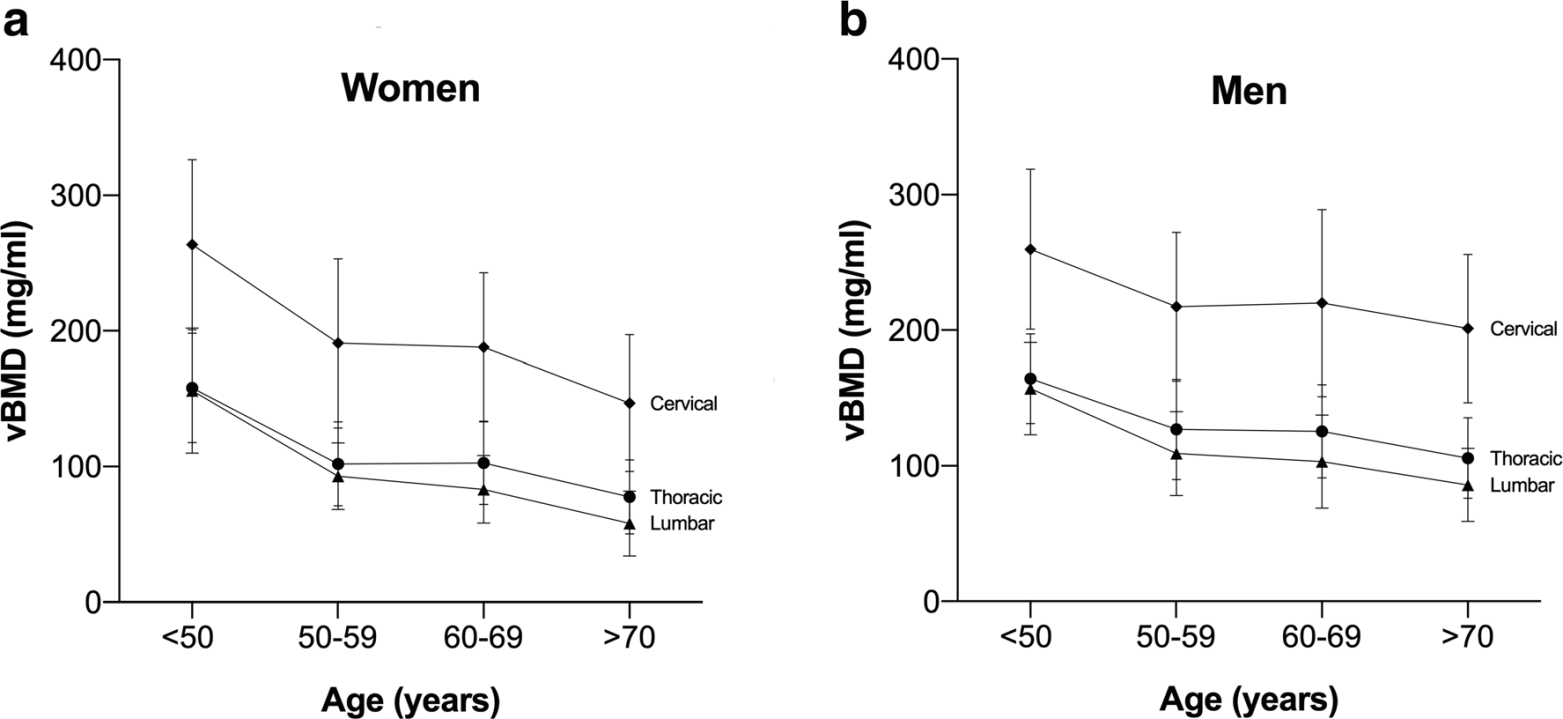
Association between age and vBMD for the cervical, thoracic, and lumbar spine in women (left) and men (right). Data points and error bars represent the respective mean and standard deviation

**Fig. 4:**
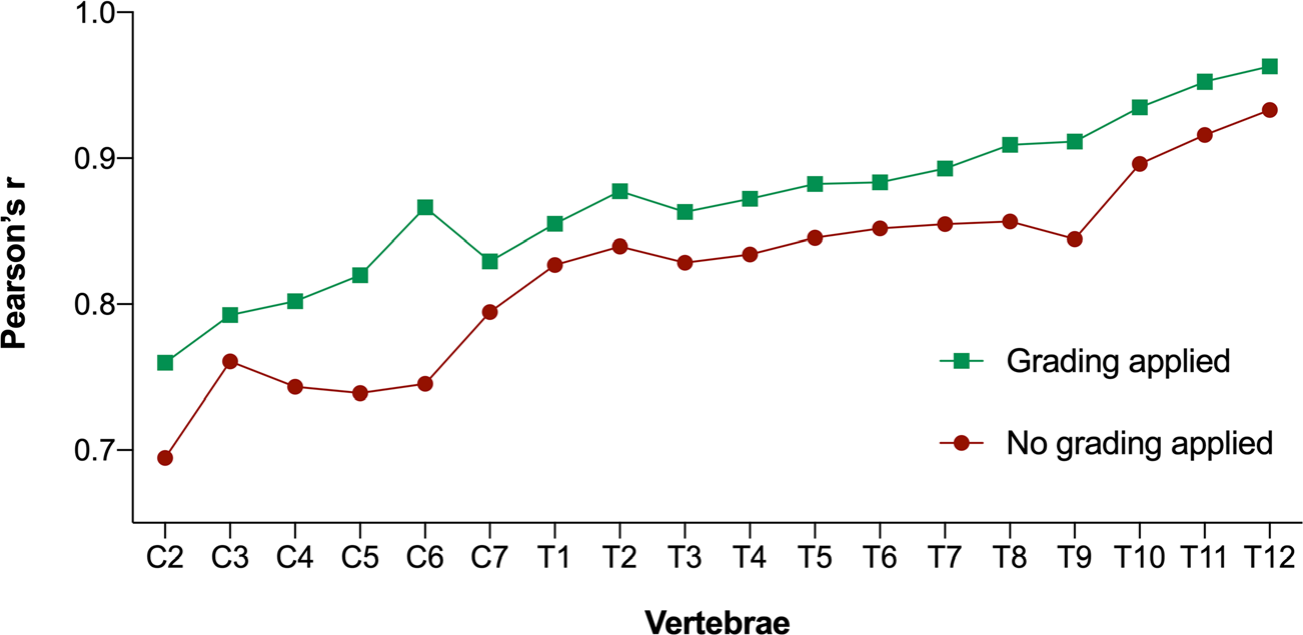
Plot showing Pearson’s correlation coefficients between vBMD of C2 through C7 with respect to the averaged vBMD of L1–L3 before (green) and after (brown) exclusion of vertebrae due to fractures and degenerative changes

**Fig. 5:**
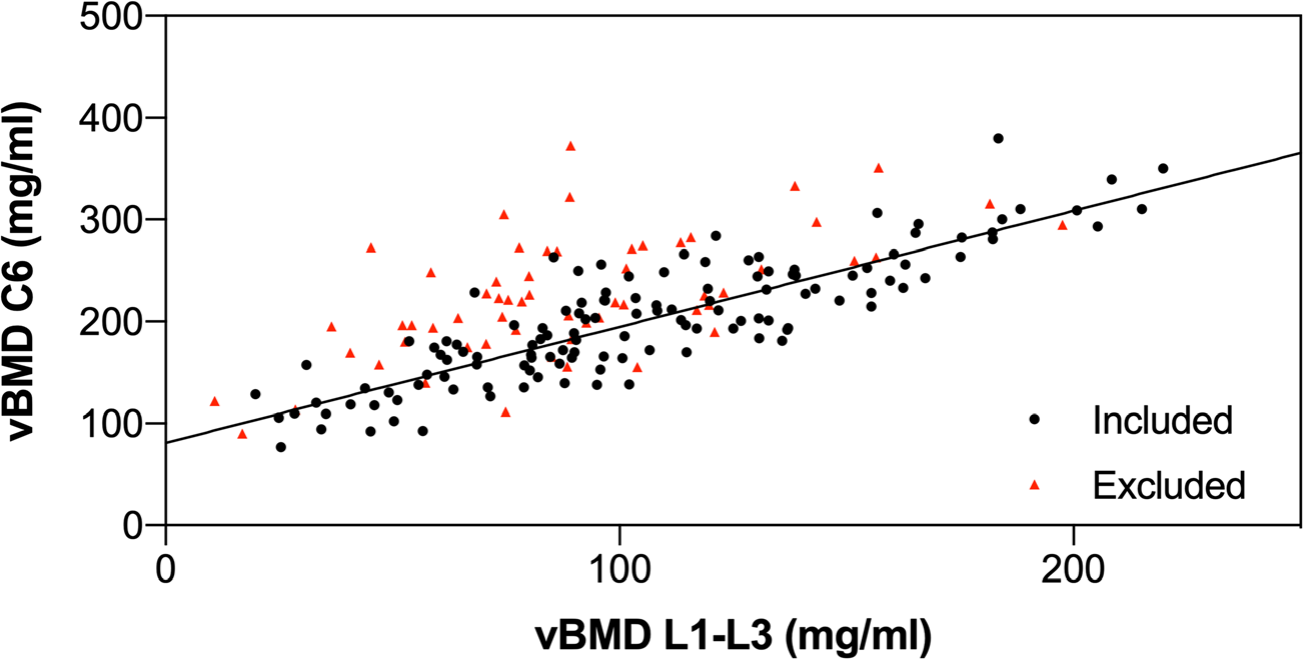
Exemplary association between cervical measurements (C6) vs. lumbar measurements (L1–L3) for vBMD. The scatterplot shows a linear correlation for the two vBMD measurements. Linear regression, *r*^2^ = 0.7504, lumbar vBMD_L1-L3_ = 1.138 × C6 + 80.98. Values from vertebrae that were excluded after a semiquantitative visual assessment based on the presence of fractures or degenerative changes are shown as red triangles

Figure 2 shows the distribution of vBMD at the spine among five different age groups. In both women and men, trabecular vBMD decreased with age for the cervical, thoracic, and lumbar regions (Fig. 3). The vBMD at all cervicothoracic levels strongly correlated with the averaged lumbar vBMD values at L1–L3, with a median Pearson’s correlation coefficient of *r* = 0.87 (range: *r*_C2_ = 0.76 to *r*_T12_ = 0.96) (Fig. 4). When not excluding fractured and degenerated vertebrae (Genant grades 1–3; moderate to severe degenerative changes, grades 2–3), the correlation decreased significantly (*p* < 0.0001) to a median Pearson’s correlation coefficient value of *r* = 0.82 (range: *r*_C2_ = 0.69 to *r*_T12_ = 0.93). The greatest decrease in correlation was observed at the C6 level (*r* = 0.87 vs. *r* = 0.75), and single data points for this relationship are shown in Fig. 5 (see the supplementary material for scatterplots of all other levels C2–T12).

**Fig. 6:**
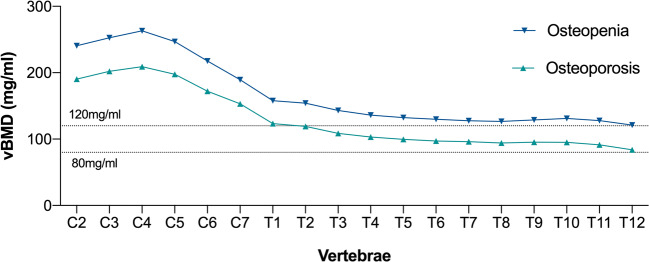
Calculated cut-off values (vBMD) for each vertebra. The dotted lines indicate the lumbar vBMD cut-off for osteoporosis (vBMD < 80 mg/ml) and osteopenia (vBMD < 120 mg/ml) as defined by the American College of Radiology

Linear regression fits were calculated to obtain cut-off vBMD values for the diagnosis of osteoporosis and osteopenia for each vertebra of the cervicothoracic spine (Fig. 6). The cut-off values for osteoporosis peaked at C4 (209.2 mg/ml) and decreased to 83.8 mg/ml at T12. Regarding the absolute cut-off values for osteoporosis and osteopenia, linear regression equations and coefficients of determination are shown in Table 2.

**Table 2:** Coefficients of determination (*r*^2^), linear regression equations, and vBMD thresholds for osteoporosis and osteopenia for each vertebra (C2–T12) in mg/ml

	*r*^2^	Linear equation	Threshold osteopenia	Threshold osteoporosis
C2	0.5772	1.253*x + 90.15	240.5	190.4
C3	0.6282	1.266*x + 100.7	252.6	202.0
C4	0.6434	1.350*x + 101.2	263.2	209.2
C5	0.672	1.229*x + 99.36	246.8	197.7
C6	0.7504	1.138*x + 80.98	217.5	172.0
C7	0.688	0.9047*x + 80.80	189.4	153.2
T1	0.7309	0.8606*x + 54.50	157.8	123.3
T2	0.7698	0.8758*x + 48.93	154.0	119.0
T3	0.7449	0.8591*x + 39.86	143.0	108.6
T4	0.7605	0.8295*x + 36.64	136.2	103.0
T5	0.7784	0.8132*x + 34.69	132.3	99.7
T6	0.7805	0.8190*x + 31.52	129.8	97.0
T7	0.7974	0.7929*x + 32.59	127.7	96.0
T8	0.8266	0.8113*x + 29.22	126.6	94.1
T9	0.8307	0.8357*x + 28.50	128.8	95.4
T10	0.874	0.9000*x + 23.01	131.0	95.0
T11	0.9069	0.9102*x + 18.62	127.8	91.4
T12	0.9268	0.9303*x + 9.346	121.0	83.8

## Discussion

Our results confirm that vBMD is significantly higher at the cervical than at the thoracolumbar spine.

This is consistent with other studies that have found the highest BMD values at C4 and C5 [21, 31]. Furthermore, a decrease in mean vBMD was observed from the mid-cervical spine in the caudal direction, similar to previous studies [20, 21]. However, a plateau was reached at the thoracolumbar transition, in agreement with a large-cohort study by Zhang and colleagues [22].

Osteoporosis screening using opportunistic CT is widely recognized as a method to accurately and reproducibly measure vBMD [3, 6, 32–34]. Diagnostic accuracy of opportunistic volumetric BMD was shown to be significantly higher than dedicated areal BMD determined by DXA, favoring this technique [15, 16]. In addition, the application of artificial intelligence operating at low cost and without additional radiation exposure (e.g., fully automated pipelines) has unlocked the enormous potential of opportunistic use of CT data [16, 35]. Since absolute vBMD values are known to vary widely along the spine, it was uncertain whether there are any significant trends or correlations whose extraction would add additional value [20, 21, 36]. Herein, by means of an automated pipeline used for clinical routine MDCT data, we show that trabecular vBMD at the spine is indeed heterogeneous, yet strongly correlated. Based on these high correlations between lumbar and cervicothoracic vertebrae, osteoporosis screening appears to be feasible in these regions as well. We postulate adjusted cut-off values for the diagnosis of osteoporosis for the thoracic spine at 100 mg/dl and for the cervical spine at 200 mg/dl. The results also demonstrate that not only fractured but also moderately to severely degenerated vertebrae significantly increase vBMD values, alter vBMD correlations, and should therefore be excluded from further evaluation. Taken together, our study suggests that CT scans covering only the cervicothoracic spine are sufficient to diagnose osteoporosis and osteopenia, or at least guide the radiologist in a particular direction. Thus, additional dedicated imagining studies for the purpose of osteoporosis screening could be spared.

Some authors have argued that osteoporotic fractures primarily affect the lumbar and thoracolumbar spine, rendering cervical BMD measurements irrelevant [20]. While the former may be true for osteoporotic compression fractures of the vertebral body, odontoid fractures are considered osteoporotic fractures as well [37]. In such patients, assessment of vBMD is of considerable interest. We showed that cervical measurements are of great value not only locally, but also to diagnose osteoporosis. The cervicothoracic spine undergoes degenerative processes as does the lumbar spine. Here, we demonstrated that although having different absolute values, the decrease in vBMD over time behaves similarly in different spinal regions. This is of particular interest prior to cervical spine surgery (e.g., stabilization procedures such as anterior discectomy and fusion). Recently, screw loosening was shown to be associated with low vBMD after lumbar semi-rigid instrumentation, further underscoring the potential importance of cervicothoracic vBMD measurements [38].

We acknowledge limitations of our study. First, the retrospective design and the enrollment of patients exclusively administered to our department may have led to selection bias, thereby limiting the generalization of our results. Second, further studies with larger cohorts are needed to approximate vBMD thresholds to a generalizable ground truth and to investigate the diagnostic performance of the postulated thresholds for fracture prediction. The clinical utility of such opportunistic measurements needs to be assessed based on such prospective studies. Furthermore, the ethical question of whether patients should be informed about such opportunistic findings at all needs to be thoroughly discussed based on detailed numbers about the possible consequences for the individual patient.

## Conclusion

In conclusion, low bone mass may be diagnosed based on cervical and thoracic vBMD, given respective correlations with the lumbar vBMD. CT scans covering only parts of the cervicothoracic spine should therefore be integrated into the workflow of automated or semi-automated data extraction pipelines. We propose diagnostic thresholds of vBMD < 200 mg/ml for the cervical spine and < 100 mg/ml for the thoracic spine as strong indicators of osteoporosis.

## Supplementary information

ESM 1 (DOCX 1017 kb)

## Abbreviations

BMD: Bone mineral density
DXA: Dual-energy X-ray absorptiometry
MDCT: Multidetector computed tomography
MIPs: Maximum intensity projections
vBMD: Volumetric bone mineral density

## Declarations

### Guarantor

The scientific guarantor of this publication is Jan S. Kirschke.

### Statistics and biometry

One of the authors has significant statistical expertise.

### Methodology

• retrospective

• diagnostic or prognostic study

• performed at one institution (with multicenter data)

## References

[1] Pickhardt PJ, Graffy PM, Zea R (2020). Automated CT biomarkers for opportunistic prediction of future cardiovascular events and mortality in an asymptomatic screening population: a retrospective cohort study. Lancet Digit Heal.

[2] Engelke K (2017). Quantitative computed tomography—current status and new developments. J Clin Densitom.

[3] Baum T, Müller D, Dobritz M (2011). BMD measurements of the spine derived from sagittal reformations of contrast-enhanced MDCT without dedicated software. Eur J Radiol.

[4] Bauer JS, Henning TD, Müeller D (2007). Volumetric quantitative CT of the spine and hip derived from contrast-enhanced MDCT: conversion factors. AJR Am J Roentgenol.

[5] Kaesmacher J, Liebl H, Baum T, Kirschke JS (2017). Bone mineral density estimations from routine multidetector computed tomography: a comparative study of contrast and calibration effects. J Comput Assist Tomogr.

[6] Boutin RD, Lenchik L (2020). Value-added opportunistic CT: insights into osteoporosis and sarcopenia. Am J Roentgenol.

[7] Zhang J, Delzell E, Zhao H (2012). Central DXA utilization shifts from office-based to hospital-based settings among medicare beneficiaries in the wake of reimbursement changes. J Bone Miner Res.

[8] (2011) OECD Statistics. Available via https://stats.oecd.org/. Accessed 10 Sept 2021

[9] Miller PD (2016). Underdiagnoses and undertreatment of osteoporosis: the battle to be won. J Clin Endocrinol Metab.

[10] Overman RA, Farley JF, Curtis JR (2015). DXA utilization between 2006 and 2012 in commercially insured younger postmenopausal women. J Clin Densitom.

[11] Hayes BL, Curtis JR, Laster A (2010). Osteoporosis care in the United States after declines in reimbursements for DXA. J Clin Densitom.

[12] Wright NC, Looker AC, Saag KG (2014). The recent prevalence of osteoporosis and low bone mass in the United States based on bone mineral density at the femoral neck or lumbar spine. J Bone Miner Res.

[13] Svendsen OL, Hassager C, Skødt V, Christiansen C (1995). Impact of soft tissue on in vivo accuracy of bone mineral measurements in the spine, hip, and forearm: a human cadaver study. J Bone Miner Res.

[14] Adams JE (2013). Advances in bone imaging for osteoporosis. Nat Rev Endocrinol.

[15] Löffler MT, Jacob A, Valentinitsch A et al (2019) Improved prediction of incident vertebral fractures using opportunistic QCT compared to DXA. Eur Radiol 29:4980–4989 10.1007/s00330-019-06018-w PMC6682570 30790025

[16] Löffler MT, Jacob A, Scharr A et al (2021) Automatic opportunistic osteoporosis screening in routine CT: improved prediction of patients with prevalent vertebral fractures compared to DXA. Eur Radiol 31:6069–6077 10.1007/s00330-020-07655-2 PMC8270840 33507353

[17] Salzmann SN, Shirahata T, Yang J (2019). Regional bone mineral density differences measured by quantitative computed tomography: does the standard clinically used L1-L2 average correlate with the entire lumbosacral spine?. Spine J.

[18] The American College of Radiology (2018) Acr–Spr–Ssr practice parameter for the performance of musculoskeletal quantitative computed tomography (Qct) 1076 6

[19] Eckstein F, Lochmüller EM, Lill CA (2002). Bone strength at clinically relevant sites displays substantial heterogeneity and is best predicted from site-specific bone densitometry. J Bone Miner Res.

[20] Yoganandan N, Pintar FA, Stemper BD (2006). Trabecular bone density of male human cervical and lumbar vertebrae. Bone.

[21] Weishaupt D, Schweitzer ME, DiCuccio MN, Whitley PE (2001). Relationships of cervical, thoracic, and lumbar bone mineral density by quantitative CT. J Comput Assist Tomogr.

[22] Zhang Y, Zhou Z, Wu C (2016). Population-stratified analysis of bone mineral density distribution in cervical and lumbar vertebrae of Chinese from quantitative computed tomography. Korean J Radiol.

[23] Burns JE (2017) Fractures and bone density: automated detection and classification on CT images 1. 000 1–10 10.1148/radiol.2017162100 PMC5584647 28301777

[24] Rühling S, Navarro F, Sekuboyina A et al (2021) Automated detection of the contrast phase in MDCT by an artificial neural network improves the accuracy of opportunistic bone mineral density measurements. Eur Radiol 32:1465–1474 10.1007/s00330-021-08284-z PMC8831336 34687347

[25] Sekuboyina A, Rempfler M, Valentinitsch A, Menze BH, Kirschke JS (2020). Labeling vertebrae with two-dimensional reformations of multidetector CT images: an adversarial approach for incorporating prior knowledge of spine anatomy. Radiol Artif Intell.

[26] Sekuboyina A, Rempfler M, Kukačka J et al (2018) Btrfly Net: vertebrae labelling with energy-based adversarial learning of local spine prior. Lect notes Comput Sci (including Subser Lect notes Artif Intell Lect notes bioinformatics) 11073 LNCS 649–657

[27] Ronneberger O, Fischer P, Brox T (2015). U-net: convolutional networks for biomedical image segmentation. Lect Notes Comput Sci (Including Subser Lect Notes Artif Intell Lect Notes Bioinformatics).

[28] Sekuboyina A, Husseini ME, Bayat A et al (2021) VERSE: a vertebrae labelling and segmentation benchmark for multi-detector CT images Med Image Anal 73 10.1016/j.media.2021.102166 34340104

[29] Genant HK, Wu CY, van Kuijk C, Nevitt MC (1993). Vertebral fracture assessment using a semiquantitative technique. J Bone Miner Res.

[30] Guglielmi G, Floriani I, Torri V (2005). Effect of spinal degenerative changes on volumetric bone mineral density of the central skeleton as measured by quantitative computed tomography. Acta Radiol.

[31] Yoganandan N, Pintar FA, Stemper BD et al (2006) Bone mineral density of human female cervical and lumbar spines from quantitative computed tomography. Spine (Phila Pa 1976) 31 73–76 10.1097/01.brs.0000192684.12046.93 16395180

[32] Fuggle NR, Curtis EM, Ward KA (2019). Fracture prediction, imaging and screening in osteoporosis. Nat Rev Endocrinol.

[33] Baum T, Müller D, Dobritz M (2012). Converted lumbar BMD values derived from sagittal reformations of contrast-enhanced MDCT predict incidental osteoporotic vertebral fractures. Calcif Tissue Int.

[34] Engelke K, Lang T, Khosla S (2015). Clinical use of quantitative computed tomography-based advanced techniques in the management of osteoporosis in adults: the 2015 ISCD official positions-part III. J Clin Densitom.

[35] Löffler MT, Sekuboyina A, Jacob A (2020). A vertebral segmentation dataset with fracture grading. Radiol Artif Intell.

[36] Anderst WJ, West T, Donaldson WF, Lee JY (2017). Cervical spine bone density in young healthy adults as a function of sex, vertebral level and anatomic location. Eur Spine J.

[37] Kaesmacher J, Schweizer C, Valentinitsch A (2017). Osteoporosis is the most important risk factor for odontoid fractures in the elderly. J Bone Miner Res.

[38] Löffler MT, Sollmann N, Burian E (2021). Opportunistic osteoporosis screening reveals low bone density in patients with screw loosening after lumbar semi-rigid instrumentation: a case-control study. Front Endocrinol (Lausanne).

